# Transarterial Radioembolization for Management of Hepatocellular Carcinoma

**DOI:** 10.1093/oncolo/oyad327

**Published:** 2023-12-21

**Authors:** Wali Badar, Qian Yu, Mikin Patel, Osman Ahmed

**Affiliations:** Division of Interventional Radiology, Department of Radiology, University of Illinois Hospital and Health Sciences System, Chicago, IL, USA; Division of Interventional Radiology, Department of Radiology, University of Chicago, Chicago, IL, USA; Division of Interventional Radiology, Department of Radiology, University of Chicago, Chicago, IL, USA; Division of Interventional Radiology, Department of Radiology, University of Chicago, Chicago, IL, USA

**Keywords:** transarterial radioembolization, yttrium-90, hepatocellular carcinoma, radiation

## Abstract

Transarterial radioembolization (TARE) with Yttrium-90 (Y90) is a growing area of study due to its benefits in early-, intermediate-, and late-stage hepatocellular carcinoma. Treatment intent, including curative therapy, bridging to transplant, and downstaging disease, informs treatment approach and dosimetry goals. Radiation lobectomy (RL) and radiation segmentectomy (RS) are the 2 main forms of Y90 administration which have shown improved survival outcomes with the development of personalized dosimetry. RS aims to achieve complete pathological necrosis with dose escalation and RL aims for local disease control as well as induction of contralateral lobe hypertrophy to improve hepatic reserve. Furthermore, TARE has been validated in head-to-head comparison to other locoregional and systemic therapies. Lastly, early potential exists for combination therapy between TARE and immune checkpoint inhibitors for advanced stage disease.

## Teaser

This review discusses the technical considerations and the role of transarterial radioembolization for management of hepatocellular carcinoma.

**Implications for Practice**

This review highlights the key technical considerations and clinical applications of transarterial radioembolization (TARE) for hepatocellular carcinoma. Furthermore, the role of TARE with respect to other locoregional, systemic, and combined immunologic therapies is described.

## Introduction

Primary liver cancer accounts for the third most cancer-related deaths worldwide.^1^ In adults, about 80%-90% of primary liver cancer cases are secondary to hepatocellular carcinoma (HCC).^2^ The prognosis of HCC is grim due to the late detection of disease with an average 5-year survival rate of 18%.^3^ Treatment options depend on the disease burden, hepatic reserve, functional status, and goals of therapy. For eligible surgical candidates with resectable disease, transplantation or surgical resection may be considered.^4^ For poor surgical candidates, advanced disease, and/or unresectable disease, locoregional therapies (LRTs) can be considered.^5^ LRT options include percutaneous ablation and transarterial therapies such as transarterial chemotherapy (TACE) or transarterial radioembolization (TARE).^6^ TARE offers promise for early-, intermediate-, and late-stage HCC and is an ongoing area of research. The purpose of this review is to discuss the technical considerations as well as the role of TARE for management of HCC.

## Technical Considerations

### Device Overview

TARE is a form of transarterial therapy to selectively deliver radioactive isotopes that are conjugated to microparticles via tumor feeding arteries to the liver.^7^ The most common isotope employed in TARE is yttrium-90 (Y90). As Y90 decays into zirconium-90, a cytotoxic β particle is emitted.^8^ The mean tissue penetrance of the β particle is 2.5 mm with maximum penetrance of 11 mm.^9^ The limited tissue penetrance allows for local delivery via arteries supplying the tumor and spares the surrounding parenchyma.^10^ There are 2 commercially available devices for loading of Y90: glass microspheres also known commercially as TheraSphere (Boston Scientific, Marlborough, MA, USA) and resin microspheres which are known commercially as SIR-spheres (Sirtex Medical, Woburn, MA, USA).^11^ The glass microparticle has higher specific activity (2500 Bq) due to its porous matrix compared to resin (50 Bq) where the isotope is bound to the surface.^12^ Given the lower activity of resin, more resin spheres are typically required to reach intended dose increasing the likelihood of embolization or blood flow stasis.^13^

### Treatment Planning

**Figure 1.**
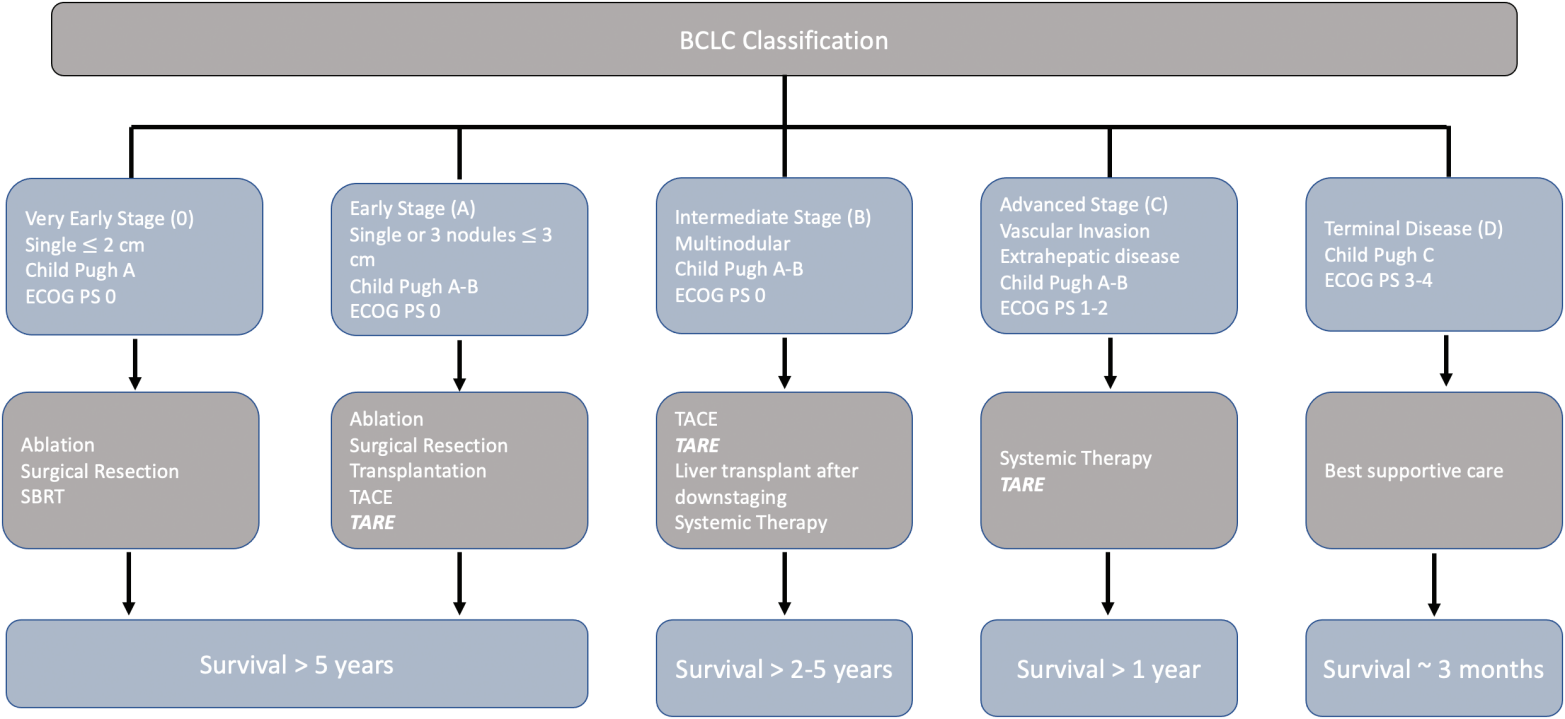
BCLC staging system for HCC. Treatment recommendations includes consensus practices. Abbreviations: BCLC, Barcelona clinic liver cancer; ECOG PS, Eastern Cognitive Oncology Group Performance Status; TARE, transarterial radioembolization; TACE, transarterial chemoembolization; SBRT, stereotactic body radiotherapy.

Prior to administration of Y90, a mapping or “planning” angiogram with Technecium-99 m macroaggregated albumin (^99m^Tc-MAA) is performed to optimize eventual Y90 delivery technique and predict dosimetry.^14^ Evolving concepts that include radiation segmentectomy (RS), radiation lobectomy (RL), or whole liver treatment can be considered based on tumor size, vascular supply, and treatment goals.^15^ RS is suitable for “curative intent” treatment of smaller lesions confined to ≤2 Couinaud’s liver segments to administer ablative doses while RL refers to high dose Y90 infusion from either the right (segments V-VIII) or left hepatic artery (segments II-IV).^16-18^ RS can be applied for curative intent as well as bridging or downstaging to liver transplantation while RL can be used for local disease control as well as bridging or downstaging to transplant.^19-23^ In addition to local disease control, RL can facilitate future tumor resection as it induces atrophy of the treated lobe and hypertrophy of the contralateral lobe with volumetric response on par with portal vein embolization.^23-26^ The application of TARE within the Barcelona Clinic Liver Cancer (BCLC) staging system is summarized in Fig. 1.

**Figure 2.**
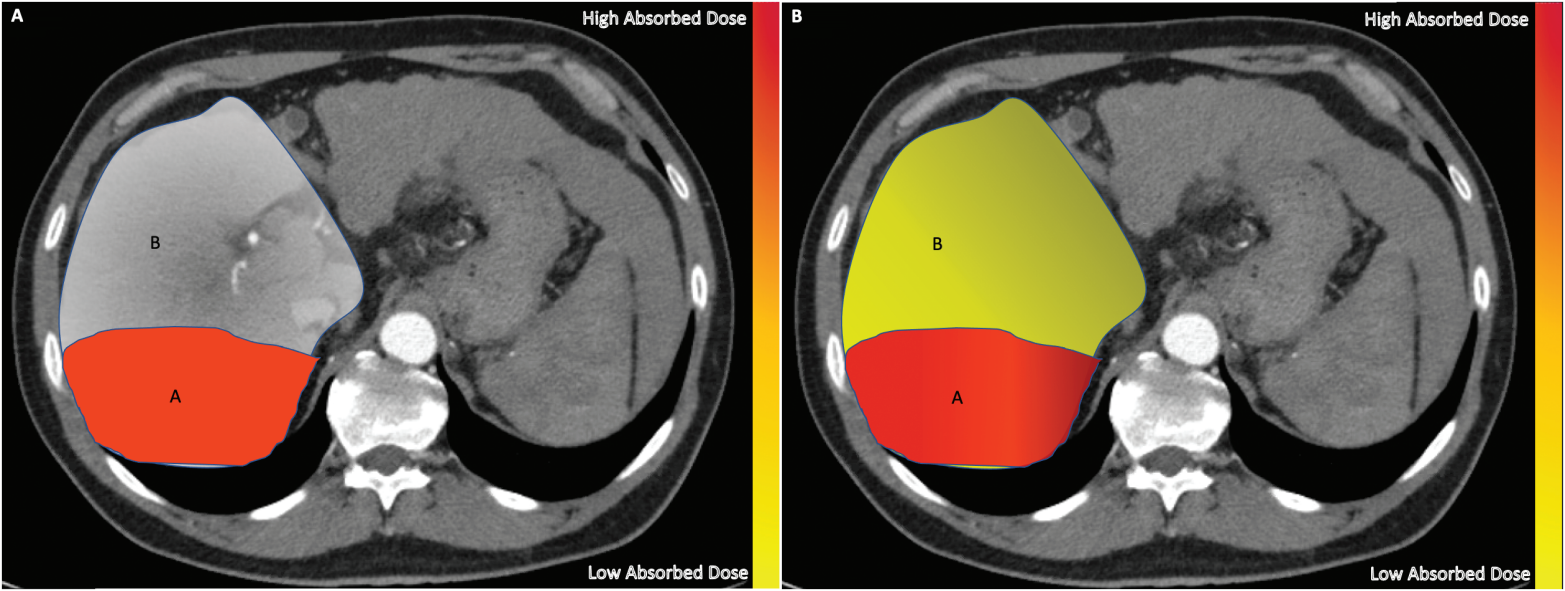
(**2A**): Contrast-enhanced CT demonstrates highlighted right hepatic lobe which contains two compartments A and B. In this radiation segmentectomy, all of the Y90 is delivered to the perfused segments VI and VII which can be modeled by the MIRD equation. No absorbed dose is seen in the non-perfused segments V and VIII (B). Absorbed dose scale is provided on left for reference with red indicating highest dose and yellow indicating lowest dose. (**B**) Contrast-enhanced CT demonstrates highlighted right hepatic lobe which contains two compartments A and B. In this radiation lobectomy, the Y90 is delivered to the two compartments per the partition equation. The tumor takes up a higher absorbed dose as expected. The nontumorous liver takes up a lower dosage as expected. Absorbed dose scale is provided on left for reference with red indicating highest dose and yellow indicating lowest dose.

Dosimetry is a growing area of interest as improved outcomes are strongly correlated with tumor absorbed dose.^27,28^ The TARGET study by Lam et al^28^ demonstrated that tumor absorbed dose for HCC was associated with improved overall survival (OS), objective response (OR), and alpha feta protein response. Personalized dosimetry utilizes quantitative methods to predict tumor absorbed dose. The most common mathematic models include the body surface area (BSA) method, the Medical Internal Radiation Dose (MIRD) model, and the partition model.^29-31^ All three of these models account for the lung shunt fraction which is determined during the MAA mapping procedure. The maximum single treatment dose to the lungs is ≤30 Gy and ≤50 Gy over multiple treatments to reduce risk of radiation pneumonitis. The MIRD and BSA assume a single compartment and do not differentiate for preferential uptake within tumor. The MIRD model is preferred for glass particles while the BSA model is used with resin particles.^10^ The partition model is applicable with both devices and considers distribution within the nontumorous liver, tumor, and lung.^10^ The partition model includes a tumor-to-normal ratio (T/N) which is determined from MAA distribution and allows clinicians to determine the highest dose deliverable to tumor while maintaining an acceptable dose within the normal liver parenchyma.^31^ Commercially available dosimetry tools such as MIM (MIM Software, Beachwood, Ohio) use voxel-based computations to create 3D models of radiotracer distribution and have shown good correlation between MAA and Y90 distribution.^15,32,33^ The partition model vs single compartment model has been studied with HCC lesions >7 cm in the DOSISPHERE, a randomized phase II, study which showed that the partition model resulted in better OR and progression-free survival (PFS) with fewer adverse events.^34^ Pictorial representations of the single compartment MIRD model and the partition model is seen in Fig. 2.

**Figure 3.**
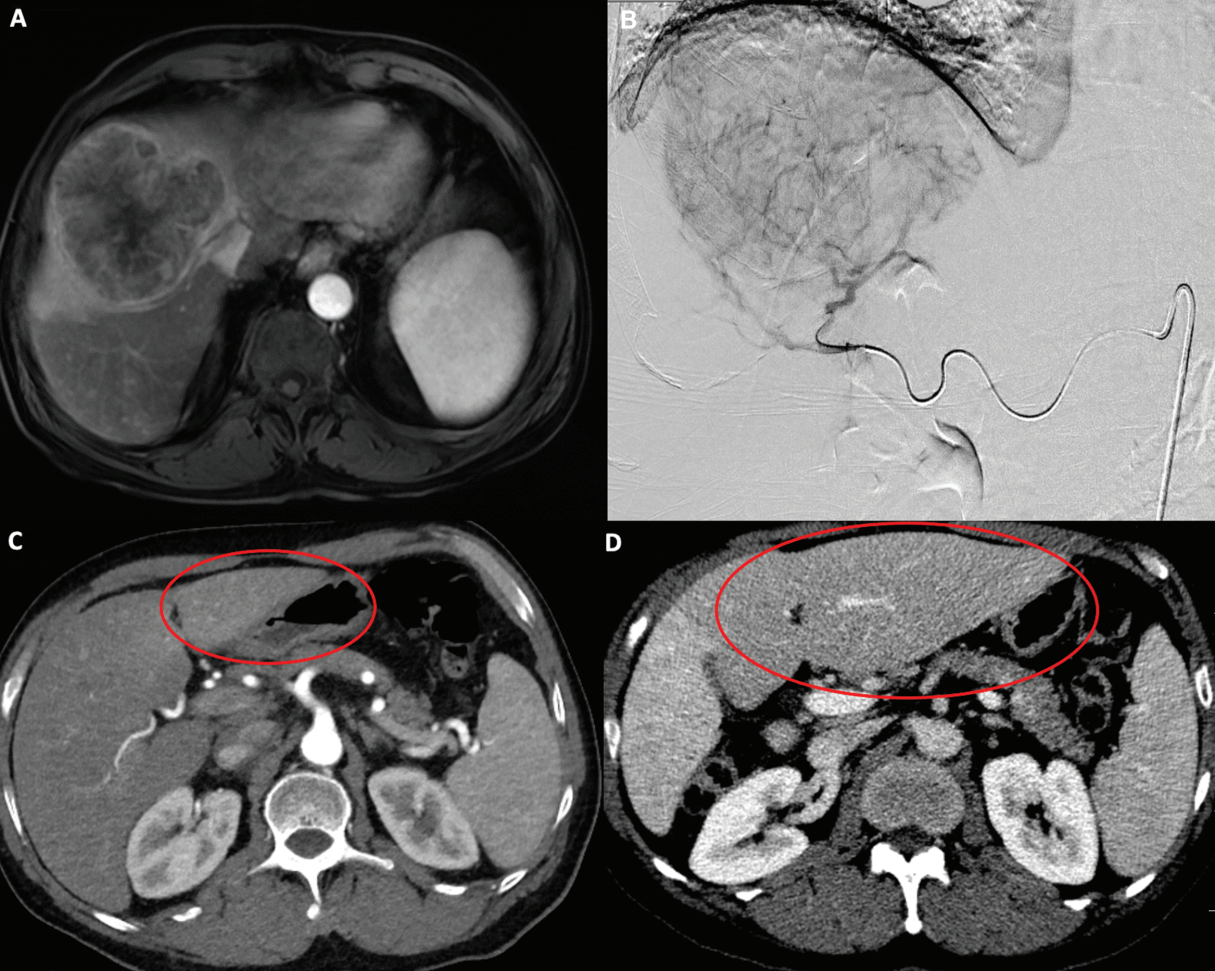
(**A**) Contrast-enhanced MRI demonstrated a large 6.9 cm segment V and VIII right hepatic lobe HCC. (**B**) Intraprocedural digital subtraction angiogram demonstrating hypervascular HCC mass to be treated with right hepatic artery radiation lobectomy. (**C**) The contralateral left hepatic lobe (FLR) is circled prior to TARE. (**D**) Left hepatic lobe, FLR, is circled 9 months after TARE and demonstrates compensatory hypertrophy.

RS is usually modeled with the MIRD equation and can tolerate higher doses (≥200 Gy) as a relatively small area of liver tissue is treated.^10^ Gabr et al^35^ observed a dose-dependent relationship on pathological necrosis with treatment naïve solitary HCC lesions ≤8 cm. Specifically, >400 Gy was associated with complete pathological necrosis (CPN) which was greater than the previously described ablative dose of 190 Gy by Vouche et al.^24^ Furthermore, the authors found that tumors with extensive to complete pathological necrosis had higher recurrence free survival (RFS) compared to partial necrosis. Additionally, CPN is an independent predictor of OS and RFS in transplant patients.^36^ Montazeri et al^18^ examined 75 HCC tumors that received RS and found specific activity had the strongest correlation with CPN underscoring the importance of the number of particles administered. RL can be modeled with both the MIRD and partition equations. The goal of RL is to deliver a sufficient dose to the tumor to ensure disease control but also to the nontumorous liver to induce atrophy and contralateral lobe hypertrophy.^10^ Palard et al^37^ evaluated the relationship between radiation dose delivered to healthy liver and hypertrophy of the future liver remnant (FLR) and found a dose of ≥88 Gy resulted in a ≥10% hypertrophy of the FLR. An example of RL and associated contralateral lobe hypertrophy is summarized in Fig. 3. Modified RL is an emerging technique in which RS is first performed to achieve CPN followed by RL to allow for downstaging and or future resection.^38^

## Overview of HCC

### TARE as Primary Therapy

TARE has been studied as primary therapy in early-, intermediate-, and late-stage HCC.^39-41^ Lewandowski et al^42^ evaluated TARE in early-stage disease with solitary tumors ≤5 cm and found that TARE was equivalent to curative-intent approaches such as radiofrequency ablation, resection, and transplantation in terms of OS and TTP for lesions ≤3 cm. In the 2021 LEGACY trial, a retrospective single arm study, Salem et al^39^ evaluated TARE as primary therapy for treatment naïve solitary HCC lesions ≤8 cm. The authors observed an OR rate of 88.3% with 62.2% of lesions exhibiting a duration of response ≥6 months. Furthermore, the 3-year OS was 86.6% and 92.8% for those who received surgical resection or transplant.

For intermediate stage disease, TACE has historically been transarterial treatment of choice.^5^ In the 2016 PREMIERE trial, a randomized phase II study, Salem et al^43^ compared TARE to TACE for BCLC stages A and B HCC and found no difference in OS but improvement in TTP with TARE with fewer adverse events. Although there was no difference in OS, TARE offered utility with better tumor control which can maintain transplant eligibility. The authors of this trial also found that TARE was associated with improved quality of life compared with TACE.^44^ In the 2022 TRACE trial, a randomized phase II study, Dhondt et al^45^ compared TARE to drug eluting beads (DEB) TACE and found TARE had improved TTP and OS with no difference in adverse events.

### TARE vs Systemic Therapy

Advanced stage disease includes vascular invasion and metastatic disease and is traditionally managed by systemic therapy.^46^ TACE is typically contraindicated for advanced disease with vascular invasion due to its embolic nature at the macrovascular level which can lead to unintended hepatic infarction.^47^ The SARAH and SIRveNIB are randomized multicenter trials investigating TARE vs sorafenib, a tyrosine kinase inhibitor.^48,49^ In the 2017 SARAH trial, Vilgrain et al^48^ compared the safety and efficacy of TARE vs sorafenib in advanced HCC and found no difference in OS or PFS but significantly better treatment tolerance with TARE. Furthermore, treatment-related adverse events were twice as frequent with sorafenib. Additionally, subset analysis in lesions previously unsuccessfully treated with TACE showed improved tumor response and quality of life with TARE. In the 2018 SIRveNIB trial, Chow et al^49^ studied the safety and efficacy of TARE vs sorafenib in locally advanced HCC and found no difference in OS, TTP, PFS but overall better tolerability with TARE. The SORAMIC trial compared TARE plus sorafenib to only sorafenib and observed to significant difference in OS but a higher adverse event rate in TARE plus sorafenib group.^50^ With the advent of personalized dosimetry and multicompartmental modeling in RL, future studies comparing optimized dosing to systemic therapies are needed.

### HCC and Combined Therapy

Systemic therapy with immune check point inhibitors have demonstrated superior efficacy compared to tyrosine kinase inhibitors which has provided an opportunity for combination therapy with TARE.^51^ The IMbrave150 study, a randomized phase III trial by Finn et al^52^, investigated atezolizumab and bevacizumab, programmed death 1 (PD-1) inhibitors, vs sorafenib for advanced HCC and found the combined therapy had superior OS, OR, PFS, and improved quality of life. In the CA209-678 trial, a phase II study, TARE was followed by nivolumab which showed a promising OR rate of 30.6%, median OS of 16.9 months, and median PFS of 3.6 months.^53^ There was a higher response rate (43.5%) in intrahepatic tumors which may suggest therapeutic synergism as TARE may help activate the immune system and promote therapeutic efficacy of PD-1 inhibitors.^54^ A Phase I/IIa trial by Lee et al^55^ investigated the therapeutic synergism of TARE followed by durvalumab for advanced HCC and observed an OR rate of 83.3%, median TTP of 15.2 months, and median PFS 6.9 months. Lastly, the GI15-225 study by McRee et al evaluated TARE followed by pembrolizumab and found an OR rate of 27%, 6-month PFS rate of 57.7%, median PFS of 8.6 months, median OS of 22 months, and median TTP of 9.9 months.^56^ While immunotherapy shows promise for combined therapy with TARE, future randomized control trials will elucidate its true incremental effect.

## Conclusion

TARE has demonstrated utility for early, intermediate, and late-stage HCC. Treatment goals include curative intent, bridging to transplant, and downsizing disease/facilitating resection which inform treatment approach (RL vs RS) and dosimetry considerations. TARE’s benefit has been validated with comparison to other LRTs and systemic therapies. Combined immunologic therapy and TARE have shown early benefit in advanced-stage HCC but future studies will guide its implementation into daily practice.

## Data Availability

No new data were generated or analyzed in support of this research.
